# Relationship between circadian rhythm-related genes and extracellular matrix: implications for sleep deprivation

**DOI:** 10.1007/s11325-023-02929-7

**Published:** 2023-11-04

**Authors:** Xuejuan Liu, Jia Sun, Zexia Ling, Tong Dong

**Affiliations:** 1 Cadre Ward of Neurology Department, Gansu Provincial People’s Hospital, Lanzhou, 730000 Gansu China; 2 Affiliated Hospital of Gansu Medical College, Pingliang, 744000 Gansu China; 3 Department of Neurology, Gansu Provincial People’s Hospital, 204 Donggang West Road, Chengguan District, Lanzhou, 730000 Gansu China

**Keywords:** Sleep deprivation (SD), ECM compositions, MMP9, Circadian rhythm, Correlation

## Abstract

**Background:**

Sleep deprivation (SD) can lead to the development of various pathological disorders. The extracellular matrix (ECM) compositions and circadian rhythm genes are two pivotal variables of SD. However, their relationships remain undefined during SD.

**Methods:**

A mouse SD model was established using a modified multiplatform water environment method. The expression of nerve growth factor (NGF) in mouse hippocampus was detected by an immunofluorescence (IF) method. Protein expression was assessed by western blot, and mRNA analysis was performed by quantitative real-time PCR (qRT-PCR). The differentially expressed genes after SD, the genes associated with stromal score, and gene expression correlation were analyzed by bioinformatic analysis.

**Results:**

The mouse model of SD was successfully established, as evidenced by the changed morphology, increased Bax and NGF levels, and downregulated Bcl-2 in mouse hippocampus after SD. The differentially expressed genes after SD were closely associated with the ECM compositions. The ECM composition metalloproteinase 9 (MMP9) was under-expressed in mouse hippocampus after SD. The hippocampal MMP9 expression was correlated with the expression levels of circadian genes PER2, PER3, TIMELESS, FBXL3, and NFIL3. PER2 and TIMELESS were upregulated in mouse hippocampus after SD.

**Conclusion:**

The current findings suggest a correlation between ECM composition MMP9 and circadian rhythm-related genes PER2 and TIMELESS in mouse hippocampus after SD, providing a novel understanding of the disorders after SD.

**Supplementary Information:**

The online version contains supplementary material available at 10.1007/s11325-023-02929-7.

## Introduction

Sleep, a mysterious and intricate event that spans about a third of human lifetime, is essential for maintaining optimum health and performance because it possesses a restorative function to repair the body [1]. Sleep is divided into non-rapid eye movement (non-REM) state, which occurs just after sleep onset, and rapid eye movement (REM) state, which the brain enters a few hours after sleep onset and during which many beneficial functions occur [2]. Adequate sleep is of great importance to human health and life. Sleep deprivation (SD) has been recognized as a health problem and can lead to the development of various pathological disorders [3, 4]. Uncovering the molecular effects of SD is crucial for understanding the mechanism underlying SD-induced disorders and the development of targeted therapies for SD-related diseases.

The extracellular matrix (ECM), composed of an array of macromolecules including glycosaminoglycans, proteoglycans, collagens, elastin, and non-collagen, can form a complex network by the interaction of matrix components and binding cells to adhesion receptors [5]. Based on the control of degrading enzymes such as metalloproteinases (e.g. MMP9, MMP3, and MMP2), ECM remodeling is continual during normal development and pathological disorders [6]. Deregulation of ECM compositions is linked to the pathogenesis of multiple diseases, such as aggressive cancer and fibrosis [7, 8]. There is strong evidence that ECM deregulation frequently occurs after SD. In a rat model of REM SD, ECM compositions MMP9 and MMP3 are markedly under-expressed in the hippocampi [9]. Furthermore, MMP9 mRNA expression is downregulated in rat cerebral cortex after SD [10].

The circadian rhythm plays an essential role in maintaining homeostasis. The circadian rhythm is controlled by many “period” genes including the Clock genes, maintaining a 24-h rhythm [11]. The circadian genes are implicated in various pathophysiological conditions, and their deregulation can contribute to the development of human diseases, such as diabetes and cancer [12, 13]. These rhythm genes can be severely disrupted by SD [14, 15]. Abnormal expression of the circadian genes induced by SD has been found to lead to memory impairment and aggravation of Alzheimer’s disease [16, 17].

The ECM compositions and circadian genes are two pivotal variables of SD. However, their relationship during SD remains to be defined. In this study using bioinformatics and SD mouse model experiments, we conducted further exploration on the relationship of the ECM compositions and circadian genes in SD.

## Materials and methods

### Establishment of a mouse model of SD

With an approved protocol by Animal Care and Use Committee at Gansu Provincial People’s Hospital, animal studies were carried out using 12 male BLAB/c mice age-matched between 6 and 7 weeks (GemPharmatech, Jiangsu, China). All mouse handling and experiments were compliant with international guidelines. All mice were housed in conventional conditions of environmental temperature (22–25°C) and relative humidity (45–60%) under a 12-h light/12-h dark cycle and given with food and water ad libitum. The mice were divided into the control group and SD model group after adaption for 7 days. The mice in the control group were allowed undisrupted sleep.

Through a modified multiplatform water environment method [18], a mouse model of SD was established for 5 consecutive days. Equipment containing twenty small platforms of 2 cm in diameter was placed in a water tank, and the water surface was kept at ~1.5 cm below the platform. In the SD model group, mice were placed on the platform for 21 h (14:00 pm–11:00 am the next day) and then placed in conventional cages for 3-h rest (11:00 am–14:00 pm). All mice were euthanized at the endpoint by CO_2_ overdose inhalation, and their hippocampal tissues were immediately dissected. One part of the hippocampal tissue was preserved in a refrigerator at −80°C for expression detection, and the other part was fixed in 4% paraformaldehyde for morphological analysis and nerve growth factor (NGF) evaluation.

### Histopathological analysis and immunofluorescence (IF)

Fixed hippocampal tissues were submitted for embedding, sectioning (5 μm), and hematoxylin and eosin (H&E) staining under standard protocols [18]. Briefly, after being hydrated and cleared with xylene, the sections were stained with hematoxylin and eosin solution (Beyotime, Shanghai, China). Morphological analysis was performed under a light microscope (Leica Microsystems, Wetzlar, Germany).

IF experiments of NGF were done on paraffin-embedded hippocampus sections with NGF rabbit polyclonal antibody (GB111206, Servicebio, Wuhan, China) at a dilution of 1:1500. Briefly, after hydration, sections were repaired in 10-mM sodium citrate repair buffer (pH=6.0) by boiling and followed by the blocking with 3% BSA before staining. Probing with NGF antibody was performed overnight at 4°C followed by a 50-min incubation in the dark with CY3-linked goat anti-rabbit IgG (GB21303, Servicebio) at a dilution of 1:300 and a 10-min incubation with DAPI (Servicebio) for nucleus staining. The slides were visualized under fluorescence microscopy (Eclipse Ni-U, Nikon, Lewisville, TX, USA).

### Western blot

Extracts of frozen mouse hippocampus were prepared with ProteoPrep® Protein Extraction Kit and accompanying protocols (Millipore, Molsheim, France). After quantification by BCA method (Beyotime), proteins (20 μg/lane) were resolved by SDS-polyacrylamide electrophoresis and transferred to polyvinylidene difluoride filters (Millipore). Signal detection was carried out by enhanced chemiluminescence (Thermo Fisher Scientific, Darmstadt, Germany) after probing with specific antibodies to Bax (mouse monoclonal, 60267-1-Ig, 1:10,000, Proteintech, Wuhan, China), Bcl-2 (mouse monoclonal, 68103-1-Ig, 1:5000, Proteintech), MMP9 (rabbit polyclonal, GB11132, 1:800, Servicebio), TIMP-1 (rabbit monoclonal, ab179580, 1:1000, Abcam, Cambridge, UK), PER2 (mouse monoclonal, 67513-1-Ig, 1:10,000, Proteintech), TIMELESS (rabbit polyclonal, ab229218, 1:2000, Abcam), GAPDH (rabbit polyclonal, 10494-1-AP, 1:10,000, Proteintech), and β-actin (rabbit polyclonal, GB11001, 1:1500, Servicebio). The anti-mouse or anti-rabbit IgG labelled by HRP (GB23301 or GB23303, 1:3000, Servicebio) was used as secondary antibody.

### Bioinformatics

The differentially expressed genes in the hippocampus after SD were downloaded from the GSE166831 dataset at GEO database (https://www.ncbi.nlm.nih.gov/geo/query/acc.cgi?acc=GSE166831). The computational algorithm ESTIMATE was used to evaluate the stromal and immune scores, and MCPcounter algorithm was applied to evaluate the endothelial score, in the transcriptome samples from the GSE166831 dataset. The relationships between these altered genes after SD and stromal, immune, and endothelial scores were analyzed by the WGCNA R package as reported elsewhere [19]. Gene Ontology (GO) and Kyoto Encyclopedia of Genes and Genomes (KEGG) enrichment analyses were carried out by ggplot2 package in R. Correlation of MMP9 level and the expression of several circadian rhythm-related genes in the hippocampus was analyzed using the GEPIA database (http://gepia2.cancer-pku.cn/index.html).

### Quantitative real-time PCR (qRT-PCR) for mRNA

RNA was extracted from frozen mouse hippocampus using MiniBEST RNA Extraction Kit and protocols (TaKaRa, Beijing, China). An aliquot of 2-μg RNA was applied for cDNA preparation based on the standard procedures using random hexamers and PrimeScript RT Reagent Kit (TaKaRa). Expression levels of mRNAs and the housekeeper transcript β-actin were gauged by SYBR-based qRT-PCR with specific primers (Tsingke, Beijing, China). The cycle threshold (Ct) value was recorded, and relative expression was determined by the 2^-ΔΔCt^ method. Primer details are shown in Table 1.

**Table 1:** Sequences of the primers used for qRT-PCR

Name (mouse)		Primers for PCR (5′-3′)
PER2	Forward	AACAAATCCACCGGCTACTG
	Reverse	CTCCGGTGAGACTCCTCTTG
PER3	Forward	AGAAGCTCCAGAGCATGGAA
	Reverse	TCTGTCTTCACAGGCGACAC
TIMELESS	Forward	CTTGCATGCAGAATGGAGAA
	Reverse	GCTCTCACCGAGGTTTTCAG
FBXL3	Forward	TGGCGATGTTTTGAATTTGA
	Reverse	TTTGATCAGCTCTGGGTGTG
NFIL3	Forward	CCATGGGTCCACTAGCAACT
	Reverse	GTTCGTCTTCCCCATCAGAA
β-Actin	Forward	CGATATCGCTGCGCTGGTC
	Reverse	AGGTGTGGTGCCAGATCTTC

### Statistical analysis

All assays were carried out at least three independent replicates. The two-tailed Student’s *t*-test (unpaired) was used to determine significance, which was evaluated by calculating *P* value (*P* < 0.05 was defined as significant). Error bars represented the standard deviation (SD). *P < 0.05, **P < 0.01, and ***P < 0.001.

## Results

### Bax and NGF are upregulated and Bcl-2 is under-expressed in the hippocampal tissues of sleep-deprived mice

**Fig. 1:**
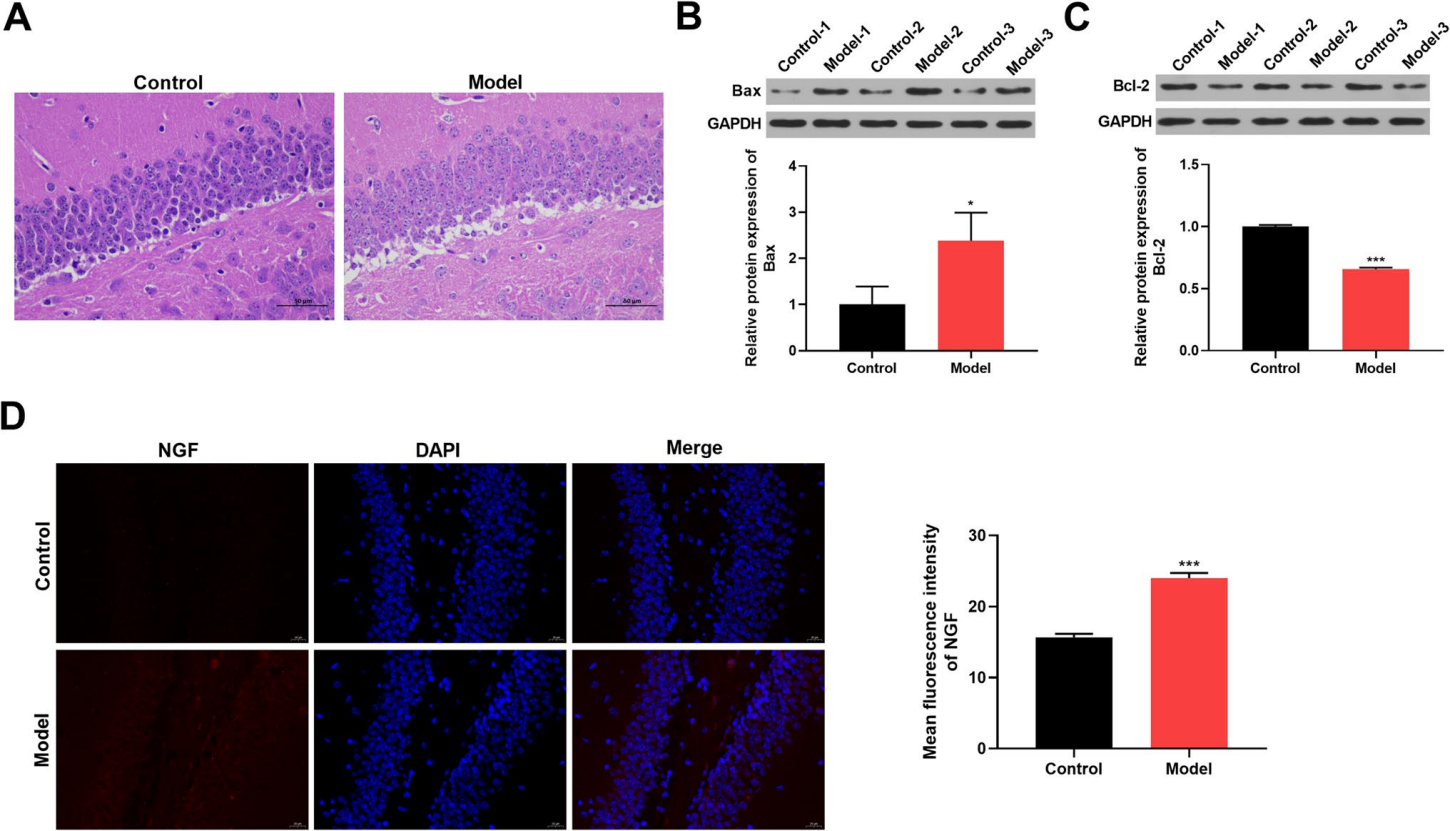
Enhancement of apoptosis and NGF expression in the hippocampal tissues of sleep-deprived mice. The mouse model of SD was generated by a modified multiplatform water environment method. The control group included six mice (*n* = 6), and the model group included six mice (*n* = 6). **A** H&E staining revealing the histopathological changes of the hippocampal tissues of sleep-deprived mice and control mice. **B**, **C** Western blot of the levels of Bax (**B**) and Bcl-2 (**C**) in mouse hippocampal tissues. **D** Representative IF assays depicting NGF expression in mouse hippocampal tissues. *P < 0.05 and ***P < 0.001

Six mice were included in the experimental group and six mice in the control group. H&E staining showed that the neurons of the hippocampal tissues of control mice were tightly and organically arranged, while the neurons of the SD model mice exhibited an atrophic body, irregular morphology, and sparse arrangement (Fig. 1A). To assess the influence on tissue apoptosis, we used western blot to detect apoptosis-related proteins Bax and Bcl-2. By contrast, the pro-apoptotic protein Bax was highly expressed and the anti-apoptotic factor Bcl-2 was under-expressed in the hippocampal tissues of sleep-deprived mice (Fig. 1B and C). IF analyses performed on hippocampal tissues revealed that NGF expression was markedly enhanced in sleep-deprived mice compared with control mice (Fig. 1D). All these data confirmed the successful establishment of the mouse model of SD.

### Association between ECM compositions and SD

**Fig. 2:**
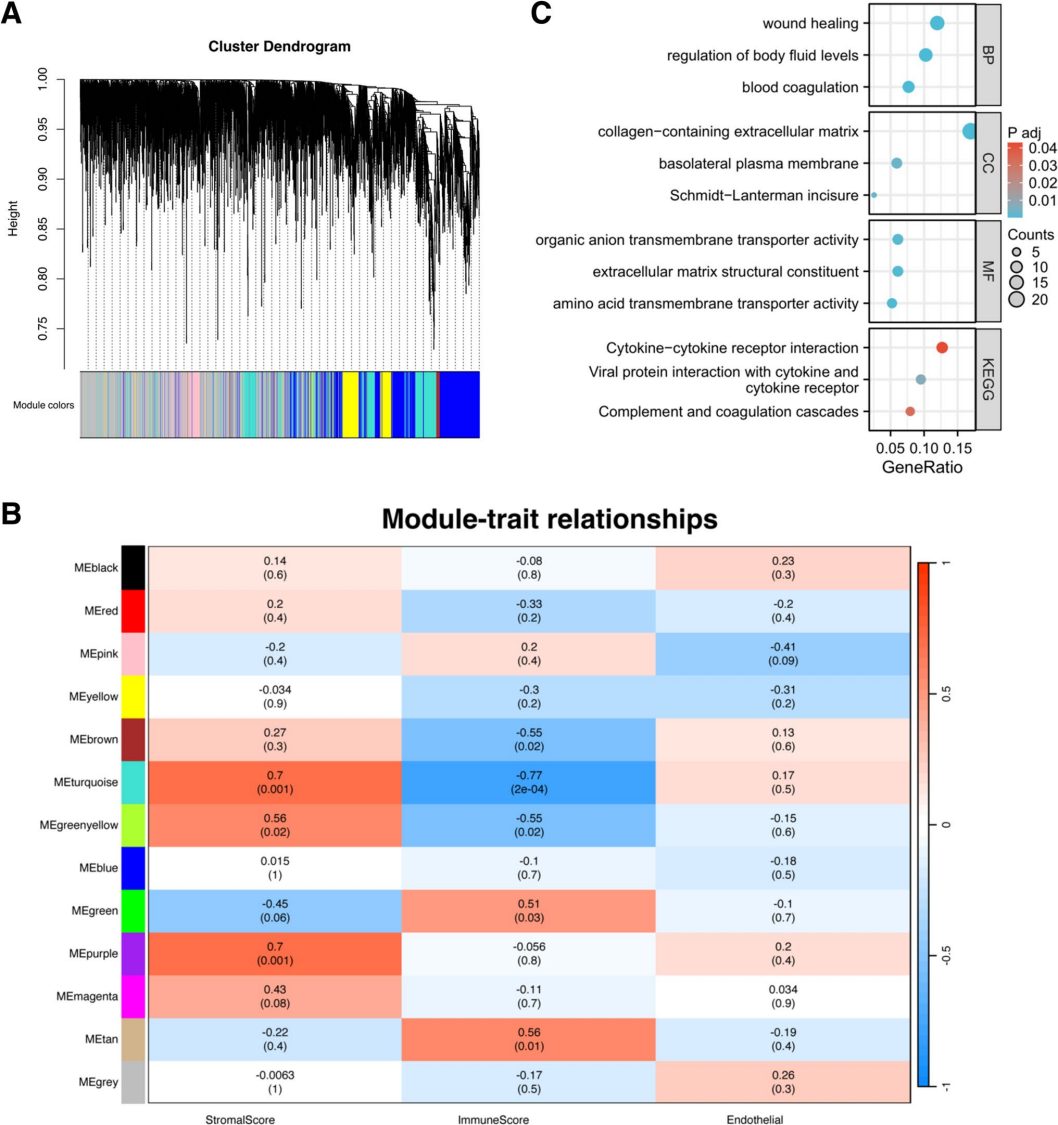
ECM compositions were tightly associated with SD. **A** WGCNA analysis of the changed genes after SD on GSE166831 dataset. Branches with different colors correspond to 13 different modules. **B** Association analysis of the genes in co-expression modules with stromal, immune, and endothelial scores. Numerical value in the module: Pearson’s correlation coefficient (*R*); numerical value within parentheses: *P* value. **C** The bubble plot revealing the significant relationship between the genes in the purple module and ECM compositions

To analyze the altered transcriptome organization following SD, the genes that were differentially expressed in the hippocampus after SD were downloaded from the GSE166831 dataset at GEO database (https://www.ncbi.nlm.nih.gov/geo/query/acc.cgi?acc=GSE166831) based on the high-throughput sequencing of C57BL/6J mice. Apart from ECM homeostasis, immune system activation and endothelial function were remarkably disturbed after SD, resulting in various disorders [20, 21]. By using two computational algorithms ESTIMATE and MCPcounter, we analyzed the stromal, immune, and endothelial scores of the transcriptome samples in the GSE166831 dataset after SD. As a result, these genes were used to construct the co-expression modules associated with stromal, immune, and endothelial scores by WGCNA. Through the power value of ten to satisfy the precondition of scale-free network distribution, 13 gene co-expression modules were identified by dynamicMods (Fig. 2A and B and Supplementary Table 1). The genes in the gray module were not co-expressed with other genes and thus could not be assigned to any module and had no reference significance. Of note, the purple module was only significantly correlated with stromal score after SD (*R* = 0.7, *P* = 0.001) (Fig. 2B). GO and KEGG pathway enrichment analyses of these genes in the purple module showed that they had a close relationship with ECM compositions (Fig. 2C).

### MMP9 was downregulated in the hippocampal tissues of sleep-deprived mice

**Fig. 3:**
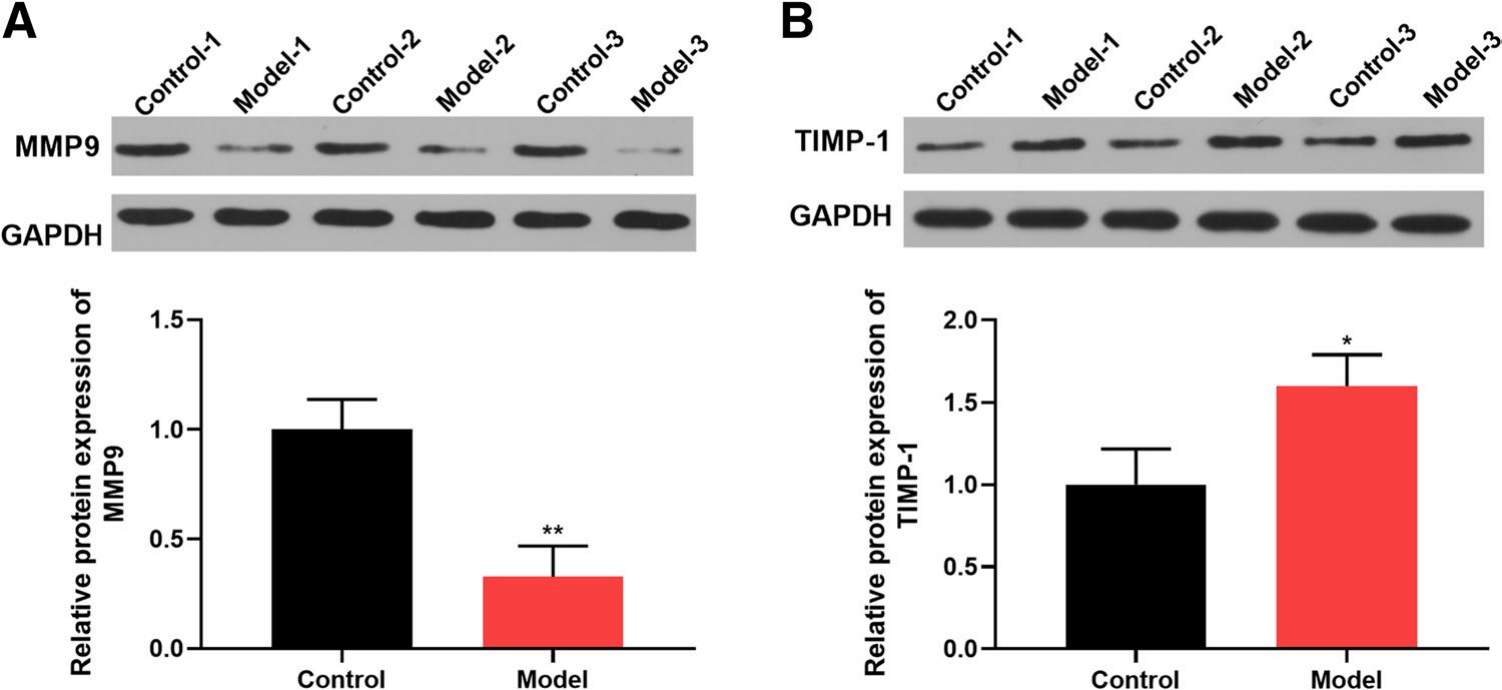
Downregulation of MMP9 in the hippocampal tissues of SD mice. **A**, **B** Western blot analyzed the expression levels of MMP9 (**A**) and TIMP-1 (**B**) in hippocampal tissues of SD mice (*n* = 6) and control mice (*n* = 6). *P < 0.05 and **P < 0.01

MMP9 induced ECM degradation and thus had the capacity to maintain ECM homeostasis [22]. MMP9 played an essential role in synaptic plasticity and sleep, and its dysregulation has been found after SD [10, 23]. Western blot analysis of the hippocampal tissues of mice confirmed that sleep-deprived mice exhibited reduced expression of MMP9 protein compared with control mice (Fig. 3A). In support of this finding, we also evaluated the expression of TIMP-1, a crucial inhibitor of MMP9. By contrast, the hippocampal tissues of sleep-deprived mice showed higher levels of TIMP-1 than controls (Fig. 3B). These data confirmed the under-expression of MMP9 after SD in mice.

### Circadian rhythm-related genes were associated with hippocampal MMP9 expression

**Fig. 4:**
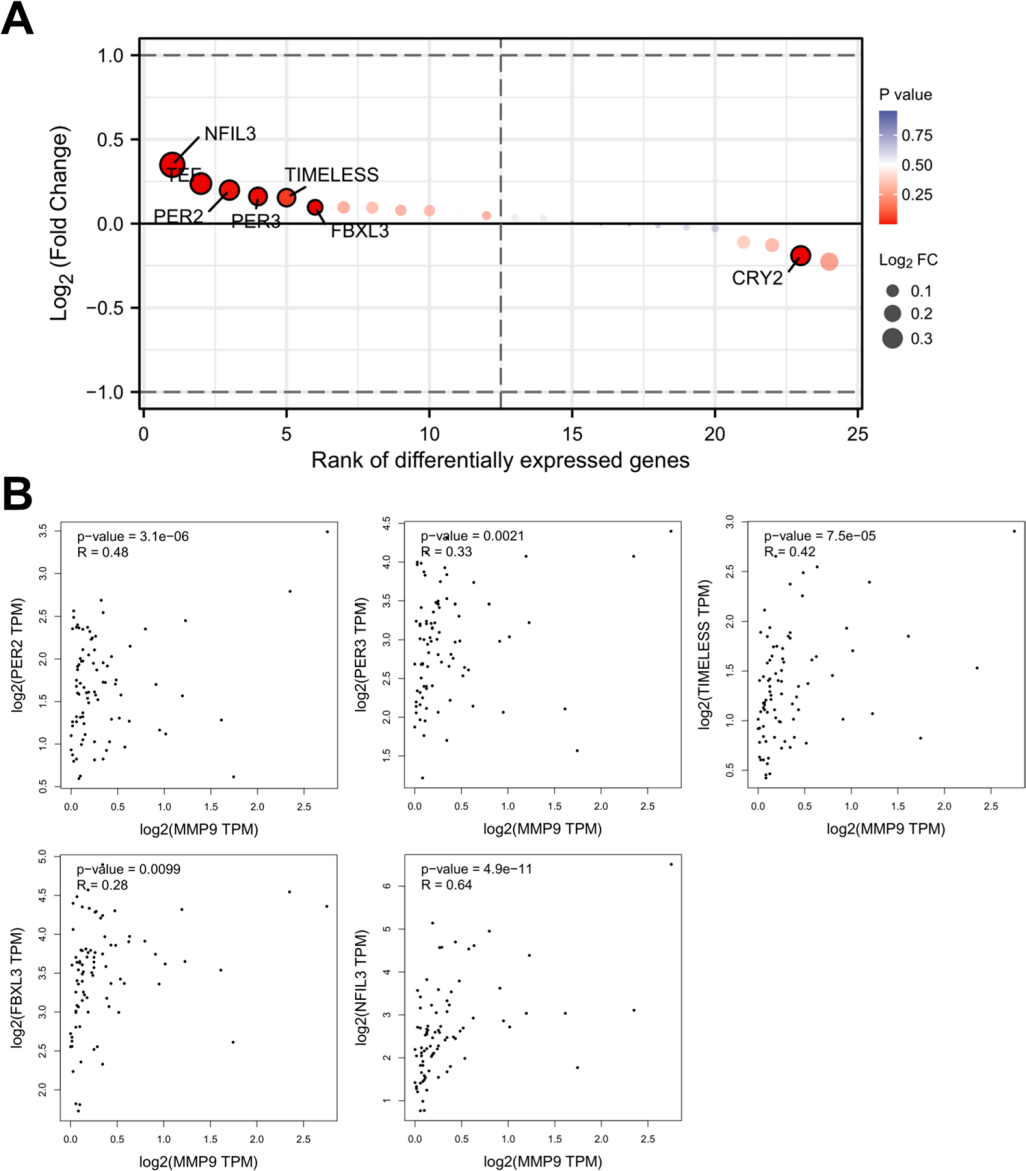
Association between circadian rhythm-related genes and MMP9 expression in the hippocampus. **A** Expression analysis of 24 circadian rhythm-related genes in the hippocampal tissues after SD. **B** Expression correlation analysis of MMP9 and PER2, PER3, TIMELESS, FBXL3, or NFIL3 in the hippocampus using GEPIA database. Pearson’s correlation coefficient (*R*)

SD induced the dysregulation of circadian rhythm genes and thus promoted the development of various disorders [16, 17]. Herein, we further observed the relationship between circadian rhythm-related genes and ECM component MMP9. When we retrieved the 24 circadian rhythm-related genes (Supplementary Table 2), we found a total of six genes (TEF, PER2, PER3, TIMELESS, FBXL3, and NFIL3) that were highly expressed in the hippocampal tissues after SD (Fig. 4A). We then used the GEPIA database (http://gepia2.cancer-pku.cn/index.html) to retrieve the expression data of the six upregulated circadian rhythm-related genes and MMP9 in the hippocampus. Correlation analyses (Pearson’s) showed that MMP9 expression was significantly associated with the levels of PER2, PER3, TIMELESS, FBXL3, and NFIL3 (Fig. 4B).

### Circadian rhythm-related genes PER2 and TIMELESS were highly expressed in the hippocampal tissues of sleep-deprived mice

**Fig. 5:**
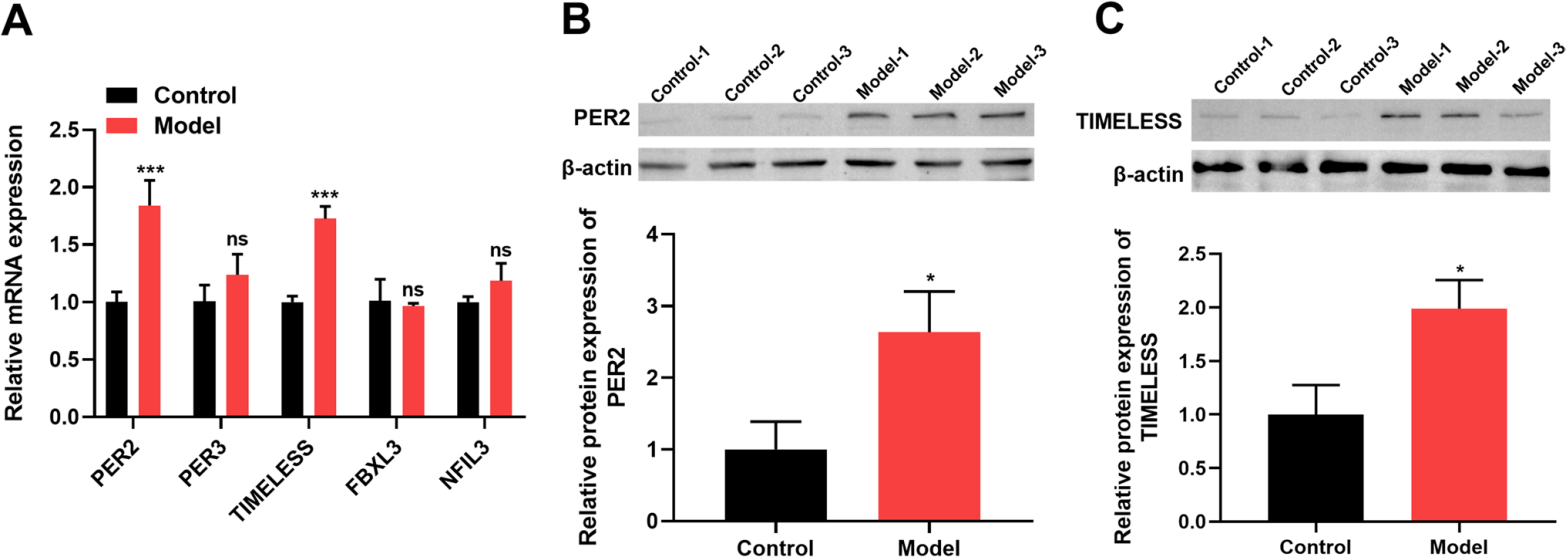
Increased expression of PER2 and TIMELESS in the hippocampal tissues of sleep-deprived mice. **A** qRT-PCR of PER2, PER3, TIMELESS, FBXL3, and NFL3 mRNAs in the hippocampal tissues of SD model mice and control mice. **B**, **C** Western blot of PER2 and TIMELESS protein levels in the hippocampus of SD model mice and control mice. *P < 0.05 and ***P < 0.001. ns, non-significant

We evaluated the expression pattern of the five circadian rhythm-related genes associated with MMP9 in the hippocampal tissues of SD mice. The data of qRT-PCR showed that the mRNA levels of PER2 and TIMELESS were highly expressed in SD mice (Fig. 5A). After SD, protein levels of PER2 and TIMELESS were strikingly enhanced in mouse hippocampal tissues (Fig. 5B and C). These observations indicated that SD had a clear effect on the expression of circadian rhythm-related genes PER2 and TIMELESS.

## Discussion

As a common disorder in modern society, SD is associated with multiple pathological and neurobehavioral problems by leading to cognitive impairment and chronic fatigue [3, 24]. For example, the human right hippocampus shows higher accumulation of β-amyloid (Aβ) after one-night of SD, suggesting that SD may be a potential risk factor for Alzheimer’s disease [25]. SD can also lead to enhanced incidence rate of cardiovascular disease by influencing the phenotypes of DNA, RNA, and protein [26]. Additionally, via the induction of hepatic lipogenic enzymes, SD contributes to steatosis and insulin resistance in mouse liver [27]. Therefore, uncovering molecular influences during SD is crucial for the development of targeted drugs to prevent and treat SD-induced diseases. In this study we first generated a mouse model of SD through the modified multiplatform water environment method, as reported previously [18, 28]. As a result, we elucidated the alterations of ECM degradation factor MMP9 and circadian genes PER2 and TIMELESS after SD in mouse hippocampus. These findings highlight the implications of circadian rhythm-related genes and ECM in SD.

Through bioinformatic analysis, we predicted the close association between ECM compositions and SD. As an intricate network in maintaining the structural and functional integrity of tissues and organs, the ECM and its remodeling play critical roles in normal development and human disease [29]. As a key part of ECM compositions, matrix metalloproteinases (MMPs) and their specific inhibitors are implicated in human pathogenesis [6]. Dysregulation of ECM composition occurs in the hippocampi after SD [9, 10]. In agreement with previous findings [9, 10], our data demonstrated the under-expression of ECM composition MMP9 and upregulation of MMP9 inhibitor TIMP-1 in mouse hippocampus after SD.

Circadian rhythm-related genes can be severely disrupted by SD and may actively participate in the treatment of SD [15, 30, 31]. Deregulated circadian genes can lead to human disorders, such as memory impairment, inflammation, and psychiatric disorders [32, 33]. Targeting circadian genes has been proposed as a rapid anti-depressant therapy after SD [34]. Numerous reports have demonstrated the relationship between circadian rhythm genes and ECM. The ECM has been reported to have the capacity to modulate intrinsic circadian gene expression in epithelial cells and fibroblasts [35, 36]. In rats with osseointegration, the ECM markers, such as Col10a1 and Col2a1, are correlated with the expression of Npas2, an ortholog of Clock [37]. During aging, the changes of ECM biochemical properties may result in the dysregulation of circadian Clock [38]. On the other hand, circadian Clock genes, such as CRY2 and Bmal1, possess critical activity in maintaining ECM homeostasis and remodeling [39, 40]. During liver disease, dysregulation of circadian gene Clock can cause fibrotic ECM deposition and thus leads to spontaneous fibrosis [41]. Although abnormal expression of circadian genes and ECM compositions occurs after SD, it is still unknown whether circadian genes are related to ECM during SD. In the present work, via bioinformatic analysis, we found the significant association between MMP9 expression and the levels of circadian rhythm-related genes PER2, PER3, TIMELESS, FBXL3, and NFL3 in the hippocampus. Furthermore, contrary to MMP9 expression, PER2 and TIMELESS, two core components of the circadian rhythm, are highly expressed in mouse hippocampus after SD. Previous work has demonstrated the expression correlation of circadian rhythm-related genes and MMP9. For instance, in HUVECs, silencing of Clock or Bmal1 can upregulate MMP9 level [42]. Conversely, Bmal1 contributes to breast cancer progression by elevating MMP9 expression [43]. Due to the small sample size, the present work is hampered by the lack of investigation into specific mechanisms of the relationship between ECM and circadian genes during SD. Further analyses will be warranted in future work. Additionally, we established the mouse model of SD using the BALB/c mice and analyzed the altered transcriptome organization after SD by GSE166831 dataset based on the C57BL/6J mice. By the high-throughput sequencing data of C57BL/6J mice after SD, we predicted the association between ECM and SD pathogenesis, which is experimentally confirmed in the BALB/c mouse model. Using two different strains of mice to elucidate the ECM-SD relationship may be more comprehensive than one mouse strain.

Collectively, the findings of the current work suggest the correlation between ECM composition MMP9 and circadian rhythm-related genes PER2 and TIMELESS in mouse hippocampus after SD, providing a novel insight into understanding the disorders after SD.

### Supplementary information

ESM 1 (XLSX 325 kb)

ESM 2 (XLSX 10 kb)

## Data Availability

All the data mentioned in this paper were displayed in the supplementary tables.
